# Supplementary material for: WordCommentsAnalyzer: A windows software tool for qualitative research
Source: F1000Res. 2018 Sep 4;7:536. Originally published 2018 May 3. [Version 2] doi: 10.12688/f1000research.14819.2 (PMC6137411; doi:10.12688/f1000research.14819.2)
Supplement: Supplementary file 3 [file f1000research-7-17614-s0002.tgz › b2a79496-4ada-47c4-9682-a99ce867836d.zip!/Eight Iranian Health Journals/Yafteh _59.docx]

© Yafteh  . This work is licensed under CC BY 4.0 (https://creativecommons.org/licenses/by/4.0/).

Volume & Issue: دوره 6 - شماره 2

Title: اثر آنتی باکتریال آلکالوئید استروئیدهای نیشکر، شوکران وعروسک پشت پرده بر روی تعدادی از باکتریهای گرم مثبت وگرم منفی

http://yafte.lums.ac.ir/article-1-913-fa.html

مقدمه: آلکالوئید ها از مواد بیولوژیکی موثری هستند که در گیاهان از جمله گیاهان داروئی وجود دارند ولذا استخراج وآزمایش اثرات ضد باکتریایی آنها اهمیت فراوانی در پزشکی، صنایع داروئی و غذایی دارد. مواد و روشها: آلکالوئید استروئید های سه گیاه مهم داروئی نیشکر، شوکران وعروسک پشت پرده استخراج واز نظر اثر مهار رشد (MIC) وکشندگی (MBC) باکتریها با روش برات میکرودایلوشن و دیسک دیفیوژن بر روی محیط کشت مولر هینتون وایزوسنسی تست آگار مورد آزمایش قرار گرفتند. یافته ها: نتایج نشان داد که آلکالوئید استروئیدهای استخراج شده از نیشکر برانتروکک فکالیس وباسیلوس سروس اثر مهار کنندگی رشد درغلظت 40=MIC وکشندگی 600 MBC= دارد. عصاره شوکران نیز در غلظت 600 MIC=MBC= یا کمتر بر اشرشیاکلی، انتروکک فکالیس استاف اپیدرمیدیس وبا سیلوس سروس موثربود. عروسک پشت پرده فقط بر باسیلوس واشرشیا کلی اثر باکتریواستاتیک وباکتریوسیدال داشت و بر باکتری های دیگر مورد آزمایش اثر باکتریوسیدی مشاهده نگردید. تقریباً هیچیک از آلکالوئید استروئیدهای مورد آزمایش بر استاف ارئوس وسودموناس ائروژینوزا اثر آنتی باکتریال نداشتند. نتیجه گیری: با توجه به نتایج فوق می توان پیشنهاد نمود تا با آزمایش بر حیوانات آزمایشگاهی اثراتin-vitro الکالوئید استروئید های گیاهان فوق را بررسی نمود

Title: میزان مقاومت آنتی بیوتیکی هلیکوباکتر پیلوری های بدست آمده از بیوپسی معده مراجعین به بیمارستان امام خمینی(ره) کرمانشاه

http://yafte.lums.ac.ir/article-1-914-fa.html

مقدمه: عفونت با هلیکوباکتر پیلوری طیف وسیعی از بیماریهای گوارشی را دربر می گیرد و در کشورهای در حال توسعه شیوع زیادی دارد. با این حال اطلاعات اندکی در خصوص میزان حساسیت آن به عوامل ضد میکروبی در دست است. هدف از این مطالعه، تعیین الگوهای حساسیت سویه های هلیکوباکتر پیلوری جدا شده از بیماران در کرمانشاه نسبت به 12 عامل ضد میکروبی متفاوت است. مواد و روشها: در این مطالعه توصیفی ـ مقطعی 72 سویه هلیکوباکتر پیلوری از بیوپسی های معده بیماران مراجعه کننده به بیمارستان امام خمینی(ره) کرمانشاه جدا شد. حداقل تراکم بازدارنده(MIC) و حساسیت این سویه ها به آموکسی سیلین، سیپروفلوکساسین، کلاریترومایسین، اریترومایسین، فورازولیدون، جنتامیسین، نالیدیکسیک اسید، نیتروفورانتوئین، مترونیدازول، پنی سیلین، ریفامپین و تتراسیکلین با استفاده از روش رقت در آگار تعیین شد. یافته ها: بر اساس نتایج به دست آمده، میزان مقاومت آموکسی سیلین 14%، سیپروفلوکساسین 42%، کلاریترومایسین 8%، اریترومایسین 10%، فورازولیدون 21%، جنتامیسین 26%، نالیدیکسیک اسید 35%، نیتروفورانتوئین 26%، مترونیدازول 34%، پنی سیلین 29%، ریفامپین 26% و تتراسیکلین 36% تعیین شد. میزان مقاومت نسبت به سیپروفلوکساسین، مترونیدازول، نالیدیکسیک اسید و تتراسیکلین در حد بالایی بود (42-34%)؛ در حالیکه در مورد فورازولیدون، جنتامیسین، نیتروفورانتوئین، پنی سیلین و ریفامپین در حد متوسط (29-21%) و در خصوص کلاریترومایسین و اریترومایسین در حد پایینی (10-8%) بود. نتیجه گیری: با توجه به نتایج فوق، سویه های مقاوم در کرمانشاه مشابه یافته های حاصل از کشورهای در حال توسعه است. لذا کشت و تعیین حساسیت جهت تعیین الگوهای مقاومت هلیکوباکترپیلوری در مناطق جغرافیایی خاص پیش از اقدام به درمان، لازم است.

Title: مقایسه تاثیر گل گاو زبان و کورتون بر بهبودی و عود درماتیت اتوپیک

http://yafte.lums.ac.ir/article-1-915-fa.html

مقدمه: درماتیت اتوپیک یک بیماری مزمن ارثی وعود کننده (در 10-5 درصد کودکان) است که درحا ل حاضر برای رفع ضایعات التهابی و خارش دار بیماران، معمولاً کورتون موضعی تجویزمیگردد. نتایج تحقیقات اخیر نشان می دهند که مبتلایان به این بیماری ، دچارکمبود اسیدهای چرب ضروری هستند و گیاه گل گاو زبان یکی از منابع غنی این ماده است که به صورت خودرو و به وفور در ایران می روید. این مطالعه به منظورمقایسه تاثیرکورتون وعصاره گل گاوزبان موضعی بر بهبودی و عود ضایعات کودکان مبتلا به درماتیت اتوپیک مراجعه کننده به درمانگاه و مطب پزشکان متخصص پوست شهر خرم آباد لرستان از بهار تا تابستان 1382 انجام گرفت. مواد و روشها: این پژوهش یک کارآزمایی بالینی است که دو گروه 25 نفره ( سن 12-0 سال) بیمار مبتلا به درماتیت اتوپیک کاملأ تصادفی انتخاب و به صورت یک در میان برای آنها کورتون موضعی یا عصاره گل گاوزبان که در اختیار همکار فارماکولوژیست قرار داشت ، تجویز شد . بعد از 10 روز دوباره بیماران توسط پزشک متخصص معاینه و از نظر شدت بیماری ، میزان بهبودی و عود ضایعات بررسی شدند. هر دو گروه 20 روز بعد از شروع درمان (10 روز بعد از اتمام مصرف دارو وعصاره) برای بار سوم معاینه واز نظر دوام تاثیر درمان ها و عود بیماری بررسی شدند . نتایج هر سه بار معاینه و سایر اطلاعات درپرسشنامه مربوطه ثبت گردید. یافته ها: یافته های این بررسی مشخص نمودکه میزان بهبودی در پاسخ به مصرف عصاره گل گاوزبان 72% خوب، 4% متوسط و 24% ضعیف بوده است. در حالیکه با مصرف کورتون 24 درصد خوب،12 درصد متوسط و 64 درصد بهبودی ضعیف داشتند. آزمون آماری X2 بین این دو گروه اختلاف معنی داری را نشان داد (003/0p<). همچنین ، ضایعات بعد از مصرف عصاره و تا 10 روز پس از قطع مصرف آن اصلأ عود نداشته؛ در حالیکه بعد از استفاده کورتون موضعی، 64 درصد عود متوسط و 36 درصد عود کم مشاهده شد. تست آماری فیشر دقیق بین میزان عود بیماری بعد از مصرف عصاره و کورتون تفاوت معنی دار ی را نشان داد (001/0p<). نتیجه گیری: به دلیل میزان بهبودی بیشتر و کم عود بودن عصاره گل گاو زبان نسبت به درمان معمول درماتیت اتوپیک، لازم است اقداماتی به منظور جایگزینی و نحوه درمان ابن بیماری صورت گیرد.

Title: بررسی وضعیت تغذیه ای کودکان 12ـ 6 ساله منطقه اسدآبادی تبریز

http://yafte.lums.ac.ir/article-1-918-fa.html

مقدمه: از آنجائیکه مقابله مؤثر با سوءتغذیه کودکان, یکی از ابزارهای دست یازیدن به توسعه پایدار بوده و شاخصهای تنسنجی از تکیهگاههای عمده در ارزیابی سلامت و بیماری در جامعه محسوب میشوند، لذا مطالعه حاضر با هدف بررسی وضعیت تغذیهای 480 کودک 12ـ6 سال (260 پسر و 220 دختر) منطقه اسدآبادی تبریز انجام گرفت. مواد و روشها: در این مطالعه توصیفی ـ مقطعی، 480 کودک سنین 12-6 سال (260 پسر و 220 دختر) به روش تصادفی نظامدار از منطقه اسدآبادی تبریز از 1000 خانوار انتخاب شد. به منظور بررسی وضعیت رشد این کودکان، قد و وزن آنها اندازه گیری شد. کودکان بر اساس نمره Z در سه گروه کم غذایی، کودکان طبیعی و کودکان مبتلا به پرغذایی تقسیم شدند. در هر سه طبقه درجات مختلف سوء تغذیه (خفیف، ملایم و شدید) تعیین شد. داده ها با استفاده از نرم افزار EPI-Info 2000 تجزیه و تحلیل شد. یافته ها: تحلیل آماری نشان داد که میزان سوءتغذیه از نوع کم غذایی برحسب نمایه قد برای سن در دختران و پسران یکسان است (18/15درصد که 38/10 و 31/11 درصد از آن به ترتیب برای پسران و دختران مربوط به کم غذایی از نوع خفیف بود). از نظر نمایه وزن برای قد, سوءتغذیه در دختران بیشتر از پسران بود (58 /34 درصد در مقابل 60/32 درصد که حدود 17/26 و 87/28 درصد از آن به ترتیب برای دختران و پسران مربوط به کم غذایی از نوع خفیف بود)، سوءتغذیه از نوع پرغذایی برحسب نمایه وزن برای قد در دختران بیشتر از پسران (41/8 درصد در مقابل 61/6 درصد)؛ ولی از نظر نمایه قد برای سن در پسران بیشتر از دختران بود (2 درصد در مقابل 74/16 درصد). نتیجه گیری: به طور کلی می توان نتیجه گرفت که در 42/40% کودکان بر اساس شاخص وزن برای قد و 23/33 درصد براساس شاخص قد برای سن، مبتلا به سوءتغذیه هستند. بنابراین در جامعه مورد مطالعه مسئله کم غذایی مشکل اصلی تغذیه ای می باشد.

Title: مقایسه عوارض بلوک اعصاب فوق ترقوه ای با دو روش تزریق معمول و

http://yafte.lums.ac.ir/article-1-919-fa.html

مقدمه: بلوک اعصاب فوق ترقوه ای یک روش متداول در جراحی های اندام فوقانی است. در این روش که معمولاً برای بلوک اعصاب سوپر اکلاویکولار انجام می شود، ماده بی حس کننده به صورت عمود بر دنده اول تزریق می گرددو می تواند با عوارضی همانند پنوموتراکس، فلج عصب فرنیک، عفونت و تزریق داخل نخاعی، تزریق داخل عروقی و سندرم هورنر همراه باشد. در این مطالعه روش تزریق با زاویه ده درجه سفالیک نسبت به دنده اول جهت کاهش این عوارض، مورد بررسی قرار گرفت. مواد و روشها: در این کارآزمایی بالینی دوسوکور، تعداد 100 بیمار که کاندید بلوک عصب ترقوه ای بودند، به طور تصادفی به دو گروه آزمایش و کنترل تقسیم شدند. برای بلوک عصب در گروه آزمایش از روش ده درجه سفالیک و در گروه کنترل از روش معمول استفاده شد. سپس عوارض دو گروه با استفاده از آزمون آماری Z مورد بررسی قرار گرفتند. یافته ها: نتایج نشان داد در گروه آزمایشی هیچ موردی از پنوموتراکس و فلج اعصاب فرنیک یافت نشد؛ در حالیکه این عوارض در گروه کنترل به ترتیب 6 و 60 درصد بود. آزمون آماری Z تفاوت معنی داری بین عوارض در دو گروه نشان داد (0001/0p<). نتیجه گیری: با توجه به نتایج این مطالعه پیشنهاد می شود این روش با سایر روشها مورد استفاده نیز مقایسه تا با اطمینان بیشتری مورد استفاده قرار گیرد.

Title: ارتباط بین آنتی اسپرم آنتی بادی ها و پارامترهای سمن

http://yafte.lums.ac.ir/article-1-920-fa.html

مقدمه: مکانیسم دقیق ایجاد ناباروری با واسطه آنتی اسپرم آنتی بادی (ASA) نامشخص است. در دستگاه تناسلی مردان ASA ممکن است اثرات زیان آوری بر بلوغ و عملکرد اسپرم یا کیفیت سمن داشته باشد. در این مطالعه در صد ASA از کلاسIgA در مردان زوجهای نابارور شهر خرم آباد بوسیله تست واکنش آگلوتیناسیون مختلط (MAR) مستقیم تعیین گردید.به علاوه پارامترهای سمن به منظور بررسی ارتباط آنها با وجود ASA مورد ارزیابی قرار گرفتند. مواد و روشها: 85 مرد بعنوان بخشی از ارزیابی ناباروری جهت تعیین ASA تست شدند. بیماران بر اساس درصد ASA 10%< یا 10% گروه بندی شدند. پارامترهای سمن (حجم کمتر از ml2، تعداد کمتر از ml/106×20 و حرکت کمتر از 50%) برای هر گروه محاسبه گردید. آنالیز آماری با استفاده از آزمون دقیق فیشر انجام گرفت. یافته ها: نتایج نشان داد که 20% جمعیت مورد مطالعه ASA مثبت بودند. حجم کمتر از ml2 با ASA به روش MAR مستقیم ارتباط معنی داری نداشت. تعداد کمتر از ml/106×20 با ASA به روش MAR مستقیم ارتباط معنی داری نداشت. حرکت کمتر از 50% بطور معنی داری با ASA به روش MAR مستقیم ارتباط معنی دار داشت (005/0 p=). نتیجه گیری: آنتی اسپرم آنتی بادی ها می توانند با آسیب به حرکت اسپرم، عملکرد طبیعی اسپرم را مختل نمایند. بنابراین پیشنهاد می گردد که بیماران با حرکت اسپرم کمتر از 50% کاندید تست ASA شوند.

Title: شیوع عوارض دیررس ختنه دانش آموزان پسر مدارس ابتدایی شهر خرم آباد در نیمسال تحصیلی 81-80

http://yafte.lums.ac.ir/article-1-922-fa.html

مقدمه: ختنه از شایعترین اعمال جراحی محسوب می شود که در تمام جهان انجام می گیرد. ختنه در صورتی که به نحو صحیحی انجام نگیرد، عوارض متعددی را در بر خواهد داشت. هدف از این مطالعه تعیین میزان عوارض دیر رس ختنه در دانش آموزان پسر مدارس ابتدائی شهر خرم آباد بود که در سال 1381-1380 انجام گرفته است. مواد و روشها: در این مطالعه مقطعی ـ توصیفی 3205 دانش آموز پسر مقطع ابتدائی در سال تحصیلی 1381-1380 از نظر عوارض دیر رس ختنه بررسی شدند. معاینه توسط کارورزان آموزش دیده رشته پزشکی، متخصص ارولوژی و پزشک عمومی به طور محرمانه در یک اتاق مجزا انجام گرفت. نکات مثبت در پرسشنامه مخصوص ثبت و بوسیله نرم افزار SPSS تجزیه و تحلیل شدند. یافته ها: نتایج بدست آمده نشان داد که 237 نفر (4/7 درصد) از مجموع 3205 نفر دچار عوارض دیر رس ختنه بودند. شایعترین عارضه، اضافه آمدن لایه داخلی پره پوس (3/39 درصد) بود. هیپوسپادیاس ختنه شده و کمبود پره پوس هر کدام با (5/2 درصد) کمترین عارضه دیررس مربوط به ختنه بودند. نتیجه گیری: با توجه به میزان بالای عوارض دیر رس ختنه در این مطالعه، برگزاری کارگاههای آموزشی برای آموزش ختنه به افرادی که در آینده انجام دهنده ختنه خواهند بود؛ از قبیل پزشکان عمومی و پرستاران و..... ضروری است. ضمنا آگاهی دادن به والدین در مورد عوارض احتمالی ختنه و تشویق آنها به انجام ختنه فرزندانشان توسط افراد متبحر جهت پیشگیری از این عوارض ضروری است.

Title: اثر ضد درد گلوکز خوراکی حین خونگیری وریدی نوزادان

http://yafte.lums.ac.ir/article-1-923-fa.html

مقدمه: مداخله های دردناک پزشکی در نوزادان بستری در بیمارستان بسیار بالاست و درمان های غیر دارویی جهت کاهش درد نوزادان هنگام مداخله های دردناک ارزشمند است. هدف از این مطالعه ارزیابی اثر ضد درد گلوگز خوراکی با غلظت متفاوت در کاهش درد نوزادان با استفاده از پاسخ های رفتاری نوزاد به تحریکات دردناک بود. مواد و روشها: این مطالعه بصورت کارآزمایی بالینی روی 65 نوزاد انجام شد. قبل از انجام مطالعه با توضیح کامل عملیات از والدین رضایت کتبی گرفته شد. هر نوزاد دو دقیقه قبل از خونگیری وریدی (3 نوبت متوالی) 3 ماده شامل آب مقطر، محلول دکستروز 30% و محلول 50% دریافت نموده و پاسخ آنها با استفاده از معیار های امتیاز بندی پاسخ های رفتاری به درد (DAN Score) اندازه گیری و ثبت گردید. مطالعه به صورت دو سو کور انجام شد و مشاهده گر از نوع ماده تجویز شده کاملاً بی اطلاع بود. نتایج با استفاده از آزمون های آماری و نرم افزار SPSS تجزیه و تحلیل شد. یافته ها: نتایج حاصل از این مطالعه نشان داد نمره درد به طور بارزی در نوزادانی که محلول شیرین دریافت کردند کمتر است. میانگین نمره درد برای آب مقطر، محلول دکستروز 30% و محلول دکستروز 50% به ترتیب 74/7، 9/6 و 48/5 بود. جنس، سن حاملگی و وزن تاثیری در پاسخ به درد نداشت. نتیجه گیری: تجویز مقدار کمی از محلول های دکستروز 30% یا 50% حین خونگیری وریدی نوزادان می تواند آثار ضد درد قابل توجهی داشته باشد. این اثرات در مورد محلول 50% بارزتر بوده و با پاسخ های رفتاری نوزادان به تحریکات دردناک قابل اندازه گیری است.

Title: تطابق بین دو شاخص مقدار عددی فشار خون و وسعت ایسکمی مغزی در

http://yafte.lums.ac.ir/article-1-924-fa.html

مقدمه: بیماریهای ایسکمیک مغزی از جمله شایعترین بیماریهای مغز و اعصاب محسوب می شوند که یکی از مهمترین عوامل زمینه ساز بروز آن پرفشاری خون (هیپرتانسیون) سیستمیک است. این مطالعه با هدف تعیین ارتباط بین دو شاخص مقدار عددی فشار خون و وسعت ایسکمی مغزی در سی تی اسکن بیماران مبتلا به ایسکمی مغزی انجام گرفت. مواد و روشها: مطالعه حاضر از نوع توصیفی ـ مقطعی بود که در نیمه اول سال 82 بر روی 66 بیمار (33 نفر زن و 33 نفر مرد) بستری در بخش نورولوژی بیمارستان سینا همدان انجام گرفت. بیماران با تشخیص ایسکمی مغزی بدون سابقه قبلی و در محدوده 45 سال و بالاتر انتخاب شدند. اطلاعات فردی، سابقه پرفشاری خون، مقادیر عددی فشار خون و مشخصات اندازه و نحوه پراکندگی ایسکمی مغزی آنان در هنگام بستری شدن از طریق سی تی اسکن اخذ و در پرسشنامه درج شدند. اطلاعات بدست آمده توسط نرم افزار EPI6 مورد تجزیه و تحلیل قرار گرفتند. یافته ها: یافته ها نشان داد که 3/63% از بیماران مبتلا در محدوده سنی 65 تا 74 سال قرار داشتند و 8/81% آنان سابقه پرفشاری خون مثبت داشتند. 3/36% از بیماران فشار خون سیستولی و 4/45% از آنان فشار خون دیاستولی طبیعی داشتند. 54 مورد (8/81%) از ایسکمی های مغزی در سی تی اسکن دارای اندازه و وسعتی بالای 2 سانتیمتر مربع بودند و 7/63% ایسکمی ها فرم متعدد داشتند. بین افزایش فشار خون سیستولی و وسعت ایسکمی ارتباط معنی داری وجود نداشت؛ ولی بین افزایش فشار خون دیاستولی و وسعت ایسکمی رابطه معنی داری مشاهده شد (03/0=P). داشتن سابقه منفی پرفشاری خون در تطابق بین دو شاخص از نظر آماری معنی دار بود (03/0=P). از نظر جنس بین دو شاخص تفاوت معنی داری وجود نداشت؛ لیکن از نظر سن در بیماران با میانگین سنی بالاتر از 75 سال این ارتباط معنی دار شود (04/0=P). نتیجه گیری: با افزایش فشار خون، وسعت ایسکمی مغزی در کسانی که سابقه پرفشاری خون قبلی ندارند، بیشتر دیده می شود و سن بالا عامل مهمی در ارتباط بین این دو شاخص است.

Title: عملکرد نوتروفیل های خون محیطی در بیماران مبتلا به تالاسمی ماژور

http://yafte.lums.ac.ir/article-1-925-fa.html

مقدمه: تالاسمی شایعترین اختلال خونی در جهان است. این کم خونی ارثی که ناشی از یک دگرگونی بنیادی در تعادل زنجیره های آلفا و بتا در ساختمان هموگلوبین بالغین (Hb.A) است, در فرم ماژور خود دارای عوارض جدی و تهدید کننده حیات (توام با همولیز و تغییرات استخوانی) است, در تحقیق حاضر عملکرد سلولهای ایمنی غیر اختصاصی (نوتروفیل ها) این بیماران مورد بررسی قرار گرفته است, همچنین ارتباط بین عملکرد نوتروفیل ها با سطح فرتینین سرم, طحال برداری, تزریق دسفرال و تعداد دفعات تزریق خون نیز مورد مطالعه قرار گرفته است. مواد و روشها: مطالعه به روش مورد ـ شاهدی روی 30 بیمار مبتلا به بتا ـ تالاسمی ماژور و 30 فرد سالم به عنوان گروه کنترل انجام شده است. برای نوتروفیلها از خون کامل حاوی ضد انعقاد هپارین (u/ml15) در حضور دکستران 6% و به کمک سانتریفوژ یخچالدار، برای جداسازی کاندیدا آلبیکنس از کلنی های قارچ روی محیط SDA و به منظور تهیه سوسپانسیونهای نوتروفیل و قارچ از محیط 1640RPMI و بافر PBS استفاده شده است. عملکرد نوتروفیلها (پس از تعیین تعداد مطلق)، با تستهای احیاء NBT، کموتاکسی، بلع، اپسونیزاسیون و کشتن داخل سلولی بررسی شد. اطلاعات حاصله به کمک آزمون های آماری t-student و X2 مورد تجزیه و تحلیل قرار گرفته اند. یافته ها: نتایج این بررسی نشان می دهدکه نوتروفیلهای بیماران مبتلا به تالاسمی ماژور از نظر تعداد و فعالیتهای بیگانه خواری (بلع)واتصال به میکروارگانیزم (اپسونیزاسیون )واحیاء NBTمشابه گروه کنترل می باشند؛ درحالیکه ازنظر قدرت حرکت هدفدار بسوی ارگانیسم (کموتاکسی )وکشتن ارگانیسم نسبت به گروه کنترل فعالیت کمتری نشان می دهند. نتیجه گیری: با انجام این تحقیق مشخص شد که بیماران مبتلا به تالاسمی ماژوردر مقایسه با گروه کنترل (سالم) حساسیت بیشتری نسبت به عفونت نشان می دهند.
